# First-pass myocardial perfusion imaging with whole ventricular coverage using L1-SPIRIT accelerated spiral trajectories

**DOI:** 10.1186/1532-429X-15-S1-P20

**Published:** 2013-01-30

**Authors:** Yang Yang, Xue Feng, Craig H Meyer, Christopher M Kramer, Michael Salerno

**Affiliations:** 1 Biomedical Engineering, University of Virginia, Charlottesville, VA, USA; 2 Radiology, University of Virginia, Charlottesville, VA, USA; 3 Medicine, University of Virginia, Charlottesville, VA, USA

## Background

First-pass perfusion imaging using cardiac magnetic resonance (CMR) has become clinically applicable as an important tool for diagnosing coronary artery disease. We have recently demonstrated that high quality first-pass images can be acquired with an optimized spiral pulse sequence, but this sequence is only capable of imaging 3 short axis slices of the heart for a maximum heart rate of 110 bpm. In order to achieve whole ventricular coverage, we have developed an accelerated spiral sequence using the Parallel Imaging and Compressed Sensing technique L1-SPIRIT.

## Methods

To evaluate and develop the accelerated reconstruction we retrospectively down-sampled data acquired from a fully-sampled variable density (VD) spiral perfusion data set acquired on a 1.5T scanner. Pulse sequence parameters included: TE 1.0 ms, TR 11 ms, FOV 320 mm^2^, in-plane resolution 1.75 mm, 8 interleaves, readout duration 8.1ms per interleave, saturation time 8 ms. We then designed a 4x accelerated pulse sequence which prospectively collected only 2 of 8 interleaves, and collects two perfusion images after each saturation pulse. Resting perfusion imaging was performed after injection of 0.1 mmol/kg of Gd-DTPA. Proton-density images acquired at the beginning of the acquisition were used to train the SPIRIT calibration kernel. Data reconstruction was performed using an iterative conjugate gradient reconstruction including a data fidelity term, SPIRIT calibration consistency term and an L1-finite difference in time as the sparsifying transform. Reconstruction was implemented in Matlab.

## Results

**Figure 1:**
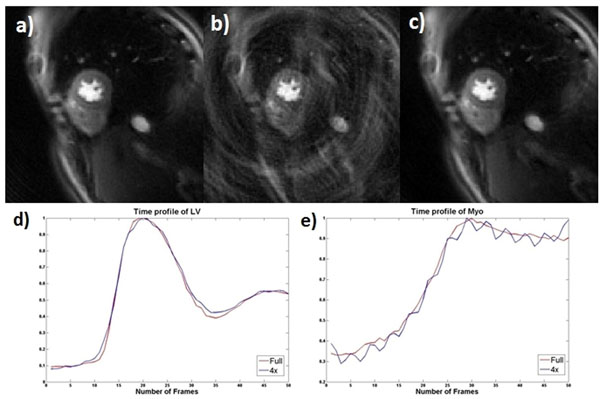
Retrospective perfusion images with fully sampled(a), 4x direct reconstruction with zero-pad (b) and the l1-SPIRiT recon (c), and temporal profiles of fully sample (red) and l1-SPIRiT (blue) from LV cavity (d) and myocardium (e)

**Figure 2:**
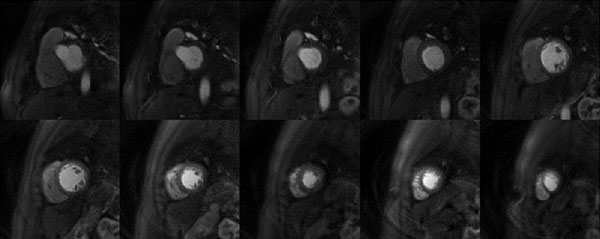
Perfusion images of 10 slices covering the whole ventricular myocardium

Figure 1 shows the fully-sampled image (a), 4x direct reconstruction with zero-pad (b) and the L1-SPIRiT accelerated results from the retrospective perfusion data. At an acceleration factor of 4, this L1-SPIRIT reconstruction resulted in excellent image quality. Temporal profiles from the LV cavity (d) and myocardium (e) were generated to assess the temporal fidelity of this reconstruction. The L1-SPIRIT curves (blue) are almost identical to the fully sampled (red) curves. The temporal oscillation on tissue function curve for the 4x accelerated images is resulting from the down sampling pattern and does not occur with actual data acquisition. Figure 2 shows perfusion images acquired with the 4x accelerated L1-SPIRIT pulse sequence which enables acquisition of 10 slices covering the whole ventricular myocardium.

## Conclusions

We demonstrated the successful application of whole ventricular coverage first-pass myocardial perfusion imaging using accelerated spirals. This sequence can acquire 8 short axis slices in 480ms enabling full ventricular coverage at heart rates up to 125 BPM. Further validation will be required in patients undergoing adenosine stress CMR.

## Funding

This work was supported by grants from AHA 10SDG2650038 and NIH K23 HL112910-01.

